# Peripheral Blood Lymphocyte-Gated Flow Cytometry Parameters and 24-Month Mortality in COPD: An Exploratory Cohort Study

**DOI:** 10.3390/jcm15145333

**Published:** 2026-07-08

**Authors:** Onur Çelik, Adil Furkan Kılıç, Konca Altınkaynak, Dursun Erol Afşin

**Affiliations:** 1 Department of Pulmonary Medicine, Erzurum Faculty of Medicine, Health Sciences University, 25240 Yakutiye, Erzurum, Türkiye; 2 Department of Internal Medicine, Faculty of Medicine, Atatürk University, 25240 Erzurum, Türkiye; adilfurkanklc@gmail.com; 3 Department of Biochemistry, Erzurum Faculty of Medicine, Health Sciences University, 25240 Yakutiye, Erzurum, Türkiye; kaltinkaynak@hotmail.com

**Keywords:** chronic obstructive pulmonary disease, flow cytometry, CD138, lymphocyte gate, mortality, biomarker

## Abstract

**Background**: Chronic obstructive pulmonary disease (COPD) is associated with substantial long-term morbidity and mortality. Peripheral blood flow cytometry may provide exploratory information regarding immune-cell distributions and activation-related markers. However, careful interpretation is required when flow cytometry outputs are derived from lymphocyte-gated percentages rather than marker-specific mean fluorescence intensity or sequential lineage-confirmed gating. We investigated whether specific lymphocyte-gated flow cytometry parameters are associated with mortality during follow-up in COPD patients. **Methods**: In this single-center observational cohort study, 51 consecutive clinically stable outpatients with COPD were enrolled in November 2023 and followed for 24 months. Baseline peripheral blood flow cytometry results were verified against archived original instrument reports. The principal exploratory flow cytometry-derived variables were CD45/SSC-defined lymphocyte-gate percentage and lymphocyte-gated CD138+ events; HLA-DR positivity was evaluated as a secondary exploratory variable. Group comparisons and descriptive receiver operating characteristic (ROC) analyses were performed. Multiplicity was assessed using a hierarchical Benjamini–Hochberg false discovery rate (FDR) framework that separated the two biologically prioritized principal variables from the remaining exploratory screening variables. For transparency, a more conservative pooled FDR correction across all ten flow cytometry-derived variables was also reported. A two-variable analysis was performed only as exploratory signal aggregation, with descriptive internal assessment using leave-one-out cross-validation (LOO-CV) and bootstrap optimism correction. **Results**: During the 24-month follow-up, 13 of 51 patients died (25.5%). In unadjusted analyses, non-survivors had lower arterial oxygen tension and nominally lower CD45/SSC-defined lymphocyte-gate percentages (median 13.08% vs. 22.63%, *p* = 0.008) and lymphocyte-gated CD138+ event percentages (median 0.07% vs. 0.39%, *p* = 0.026) than survivors. Within the hierarchical analytical-family framework, both CD45/SSC-defined lymphocyte-gate percentage and lymphocyte-gated CD138+ events retained significance in the principal-variable family (within-family q = 0.016 and 0.026), whereas no secondary-family parameter, including HLA-DR (within-family q = 0.50), did; significance was not retained under a single correction across all ten parameters (CD45 q = 0.081; CD138 q = 0.129). Descriptive AUCs were 0.749 for CD45/SSC-defined lymphocyte-gate percentage and 0.710 for CD138+ events. The two-variable signal-aggregation analysis yielded an apparent AUC of 0.858, an LOO-CV AUC of 0.796, and a bootstrap optimism-corrected AUC of 0.832. NLR was available for all 51 patients; NLR-adjusted analyses did not establish clinical incremental utility. **Conclusions**: Lower CD45/SSC-defined lymphocyte-gate percentage and lower lymphocyte-gated CD138+ event percentage showed within-cohort associations with 24-month mortality in this small COPD cohort. These observations should be regarded solely as hypothesis-generating signals. Neither principal finding was retained after pooled correction across all ten flow cytometry-derived parameters, and no incremental prognostic value beyond routine inflammatory indices or established clinical predictors was demonstrated. External validation was absent; prospective replication in larger, appropriately adjusted cohorts is required.

## 1. Introduction

Chronic obstructive pulmonary disease (COPD) is the third leading cause of death worldwide and caused approximately 3.4 million deaths in 2023 [1]. Its clinical heterogeneity and complexity present challenges for ongoing management and prognostic prediction [2]. Traditional inflammatory biomarkers such as C-reactive protein (CRP) [3] and the neutrophil-to-lymphocyte ratio (NLR) [4] are widely investigated for risk stratification in COPD. However, these markers reflect non-specific systemic inflammation and may not fully capture immune-cell alterations relevant to adverse clinical outcomes. Evidence also suggests that immune dysfunction and altered adaptive immune responses may contribute to vulnerability to infection and poor outcomes in COPD [5].

Flow cytometric analysis of peripheral blood lymphocyte subsets offers a window into the immune landscape of COPD. CD45 (PTPRC, protein tyrosine phosphatase receptor type C) is a transmembrane glycoprotein expressed across leukocytes, and CD45-versus-SSC gating is routinely used to identify lymphocyte populations in clinical flow cytometry [6]. CD138 (syndecan-1) is a marker associated with mature plasma cells [7], though its interpretation requires caution when measured as a single-parameter percentage without sequential lineage-confirmed gating. In parallel, low immunoglobulin levels have been associated with an unfavorable clinical course in hospitalized patients with COPD [8]. HLA-DR is an activation-associated marker, and increased HLA-DR expression has been reported in several T-cell subsets during acute exacerbations of COPD [9]. The interpretation of these markers depends critically on the gating strategy and on whether values represent expression intensity or gate-derived percentages.

Despite increasing interest in systemic inflammatory and immune-related biomarkers in COPD, the long-term prognostic relevance of routinely generated lymphocyte-gated flow cytometry parameters remains insufficiently characterized. In particular, it is unclear whether gate-derived lymphocyte proportions, lymphocyte-gated CD138+ events, and lymphocyte-gated HLA-DR positivity are associated with fixed long-term mortality outcomes when their measurement limitations are explicitly acknowledged. Therefore, this exploratory study aimed to evaluate the association between verified peripheral blood flow cytometry-derived lymphocyte-gated parameters and 24-month all-cause mortality in patients with COPD, while carefully distinguishing gate-derived percentages from marker expression intensity or lineage-confirmed immune-cell populations.

## 2. Methods

Study Design and Patient Enrollment: This observational single-center cohort study was conducted in the outpatient clinic of the Department of Chest Diseases at Erzurum Regional Training and Research Hospital. Consecutive clinically stable patients with COPD attending outpatient follow-up during November 2023 were screened for participation. No selective sampling strategy was applied; all eligible patients who met the study criteria and provided informed consent were enrolled consecutively. Flow cytometric immunophenotyping was performed prospectively for research purposes under the approved protocol and was not requested because of a clinical suspicion of hematologic or immunological disease. A total of 51 patients with COPD were included. COPD diagnosis was based on compatible clinical features, risk-factor exposure, and GOLD-defined spirometric confirmation, including post-bronchodilator FEV1/FVC < 0.70. Clinical stability was defined as the absence of acute exacerbation and absence of antibiotic or systemic corticosteroid treatment for respiratory worsening during the preceding four weeks, together with no COPD-related hospitalization during the preceding six weeks. Patients with current systemic corticosteroid treatment, coexisting hematologic neoplasms, interstitial lung disease, autoimmune disease, cerebrovascular or allergic diseases, or other immune-related conditions were excluded. Use of inhaled corticosteroid-containing maintenance therapy was permitted and was not an exclusion criterion. Ethics approval was obtained before enrollment began. The study was approved by the Scientific Research Ethics Committee of Health Sciences University Erzurum Faculty of Medicine (approval no. 2023/05-50; approval date: 13 September 2023), and all participants gave informed consent.

Follow-up and Outcome Definition: Vital status was assessed using hospital records and the national death registry. The primary outcome was fixed 24-month all-cause mortality. Based on 24-month survival status, the cohort was divided into survivors (alive at 24 months, n = 38) and non-survivors (died within 24 months, n = 13).

Pulmonary Function Testing and COPD Severity Variables: Baseline spirometry was performed for all patients using a MIR Spirolab III device (MIR Medical International Research S.p.A., Rome, Italy) according to the American Thoracic Society/European Respiratory Society (ATS/ERS) 2019 technical standard [10]. The patient’s age, sex, height, and weight were entered into the device, and percent-predicted values were calculated according to the Global Lung Function Initiative 2012 reference equations [11]. COPD diagnosis was based on compatible clinical features, risk-factor exposure, and GOLD-defined spirometric confirmation, including post-bronchodilator FEV1/FVC < 0.70 [12]. GOLD airflow limitation grades were assigned using FEV1 percent predicted. GOLD A/B/E groups were assigned using symptom burden and exacerbation risk, with group E defined by at least two moderate exacerbations in the previous year or at least one COPD-related hospitalization, and group B defined by mMRC ≥ 2 in patients not meeting group E criteria. Because the 6-min walk distance was not available, the full BODE index was not calculated. Instead, a partial BODE component score was derived from the available BODE components (BMI, FEV1 percent predicted, and mMRC dyspnea score) and reported only as a descriptive clinical-severity variable; it was not interpreted as the validated BODE index [13].

Laboratory Measurements: Blood samples were collected at baseline for routine laboratory tests, including complete blood count, arterial blood gas analysis, C-reactive protein, and fibrinogen levels. In addition, peripheral blood was collected in EDTA tubes for flow cytometric immunophenotyping of lymphocyte subsets and activation markers.

Immunophenotyping by Flow Cytometry and Quality Assurance: Flow cytometry was performed on fresh whole-blood samples collected in EDTA tubes and processed within 2 h of collection. Red blood cells were lysed using VersaLyse lysing solution (Beckman Coulter, Inc., Brea, CA, USA), and the remaining leukocytes were washed and stained with fluorochrome-conjugated monoclonal antibodies. The antibody panel included CD45-Krome Orange (clone J33), CD3-Pacific Blue (UCHT1), CD4-PC5 (13B8.2), CD8-FITC (SFCI21Thy2D3), CD5-PC7 (BL1a), CD19-ECD (J3–119), CD138-APC (B-A38), HLA-DR-PE (Immu-357), and CD38-APC-Alexa Fluor 750 (LS198-4-3). In a separate tube, CD26 expression was assessed using CD26-PE (4EL-1C7) (all antibodies from Beckman Coulter, Inc., Brea, CA, USA). Data acquisition was performed using a Navios flow cytometer (Beckman Coulter, Inc., Brea, CA, USA). For the tube used for CD138 analysis, a standardized total of 340,000 events was acquired. Based on the review of the archived flow cytometry reports for the study cohort, the lowest CD45/SSC-defined lymphocyte-gate percentage in the CD138-analysis tube was 5.76%, corresponding to approximately 19,584 lymphocyte-gated events. Daily instrument quality control procedures were performed before sample acquisition according to the laboratory quality assurance protocol. Unstained negative-control samples were used to establish marker-positive quadrant boundaries, including the threshold for CD138 positivity. Because CD138-positive events occurred at very low frequencies in several samples, low-positive CD138 results underwent additional gate review and re-evaluation of archived dot plots before final data recording. No repeat staining or repeat acquisition was performed for these samples.

**Figure 1:**
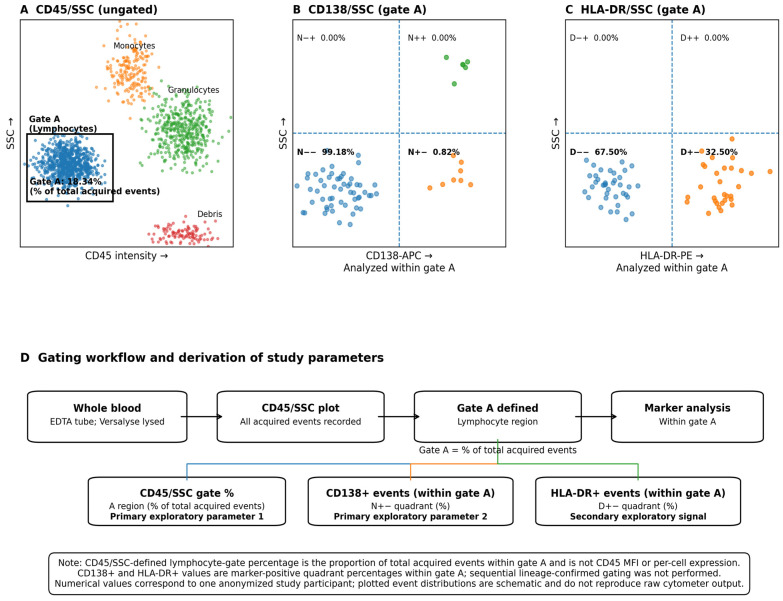
Schematic illustration of the flow cytometric gating strategy used for the derivation of the study parameters. (**A**) Lymphocytes were identified as gate A on an ungated CD45-versus-side-scatter intensity (SSC) plot. The CD45/SSC-defined lymphocyte-gate percentage represents the proportion of total acquired events located within gate A and does not reflect CD45 mean fluorescence intensity (MFI) or per-cell CD45 expression. (**B**) Within gate A, CD138 positivity was determined using a CD138-versus-SSC quadrant plot; the resulting percentage represents lymphocyte-gated CD138+ events and not a lineage-confirmed plasma-cell population. (**C**) HLA-DR positivity was similarly evaluated within gate A and interpreted as overall lymphocyte-gated HLA-DR positivity. (**D**) Workflow illustrating the derivation of the evaluated flow cytometry-based parameters from whole-blood analysis. Unstained negative controls were used to define marker-positive regions. Sequential lineage-confirmed gating was not performed. Numerical values correspond to one anonymized participant in the study cohort, whose study inclusion and sample identity were verified from source records; the plotted event distributions are schematic and are presented solely to illustrate the gating workflow rather than to reproduce raw cytometer output. Colored and dashed lines are schematic visual aids indicating gate, quadrant, and workflow boundaries; they do not represent validated numerical decision thresholds.

Gating Strategy, Variable Definitions, and Blinded Assessment: Leukocyte distribution was initially evaluated on an ungated CD45-versus-side-scatter intensity (SSC) dot plot. Based on CD45 and SSC characteristics, regional gates corresponding to lymphocytes, monocytes, granulocytes, and debris were identified. The proportion of total acquired events located within the lymphocyte region (gate A) was recorded as the CD45/SSC-defined lymphocyte-gate percentage. This parameter is a gate-derived percentage and does not represent CD45 mean fluorescence intensity (MFI) or per-cell CD45 expression. Within gate A, marker positivity was assessed using marker-versus-SSC quadrant plots, and lymphocyte-gated positive event percentages were recorded for CD3, CD4, CD5, CD8, CD19, CD26, CD38, CD138, and HLA-DR. CD138 positivity was defined using unstained negative-control-based quadrant boundaries and is reported as lymphocyte-gated CD138+ events because sequential lineage-confirmed plasma-cell gating (for example, CD19low/-CD38highCD138+) was not performed. HLA-DR positivity was measured within the overall lymphocyte-gated population and was not attributed to a specific cellular lineage. All gates were defined and reviewed by the same investigator, who was blinded to 24-month mortality status during flow cytometry assessment and data verification. During source-data verification, an incorrect date display in the archived flow cytometry report output attributable to a device/system date-setting error was documented; sample identity and the actual November 2023 study enrollment timing were confirmed from the corresponding laboratory and study records. Original instrument report outputs were retained without retrospective alteration. All flow cytometry-derived values were checked against archived original instrument reports before statistical analysis. One CD138 value that had been transcribed from the negative quadrant was corrected using the corresponding positive-quadrant value in the original report, and the verified dataset was used for all analyses. Reporting of sample handling, antibody panel, instrument, gating strategy, and control procedures was structured in accordance with established flow cytometry reporting principles [14]. The schematic gating workflow and derivation of the evaluated flow cytometry-based variables are illustrated in Figure 1.

Statistical Analysis: Statistical analyses were performed as exploratory signal-detection analyses rather than confirmatory prognostic model development. Analyses were performed using IBM SPSS Statistics version 25.0 (IBM Corp., Armonk, NY, USA) and MedCalc Statistical Software version 20.218 (MedCalc Software Ltd., Ostend, Belgium); additional sensitivity analyses were performed in Python version 3.13.5 (Python Software Foundation; https://www.python.org, accessed on 30 June 2026). Categorical variables were summarized as counts and percentages, and continuous variables were presented as mean ± standard deviation or median with interquartile range (IQR), as appropriate. Because flow cytometry-derived variables and several clinical variables were bounded or non-normally distributed, between-group comparisons for these parameters were performed using the Mann–Whitney U test. Categorical variables were compared using Fisher’s exact test when appropriate. For categorical comparisons involving more than two categories, the Fisher–Freeman–Halton exact test was used. After source verification, complete neutrophil and lymphocyte data were available for all 51 patients, so NLR was available for the full cohort (13 non-survivors and 38 survivors). GOLD airflow limitation grade, GOLD A/B/E group, and the partial BODE component score were evaluated descriptively as baseline clinical-severity variables.

The principal exploratory flow cytometry-derived variables were CD45/SSC-defined lymphocyte-gate percentage and lymphocyte-gated CD138+ events. HLA-DR positivity within the lymphocyte-gated population was evaluated as a secondary exploratory variable. Multiplicity was assessed using a hierarchical FDR framework that separated the two biologically prioritized principal variables from the remaining exploratory screening variables. This framework was used for structured exploratory interpretation, and a more conservative pooled FDR correction across all ten flow cytometry-derived parameters was also reported for transparency. The two biologically prioritized principal variables, CD45/SSC-defined lymphocyte-gate percentage and lymphocyte-gated CD138+ events, constituted the principal-variable family, within which Benjamini–Hochberg false discovery rate (FDR) adjustment was applied [15]. The remaining eight lymphocyte-gated parameters (HLA-DR positivity and the CD3, CD4, CD5, CD8, CD19, CD26, and CD38 percentages) constituted a secondary screening family, within which FDR adjustment was applied separately. Both unadjusted *p* values and within-family FDR-adjusted q values are reported. Variables reaching within-family significance in the principal-variable family were reported as the principal findings of this exploratory study; given the limited event number and single-center design, they were nonetheless considered to require prospective external replication and were not interpreted as validated clinical or technical thresholds. For transparency, a single FDR adjustment applied jointly across all ten parameters is also reported. ROC analyses were performed descriptively to estimate internal discrimination for fixed 24-month all-cause mortality, with AUCs and 95% confidence intervals estimated using the DeLong method. Internally derived cutoffs, sensitivity, specificity, and predictive values were descriptive measures only and are reported in Supplementary Table S1; they were not interpreted as validated clinical, prognostic, diagnostic, or technical thresholds.

Because only 13 mortality events occurred, multivariable modeling was restricted. A two-variable logistic regression analysis including CD45/SSC-defined lymphocyte-gate percentage and lymphocyte-gated CD138+ events was performed only as an exploratory signal-aggregation analysis and not as the development or validation of a clinical prediction model. Internal discrimination was examined descriptively using LOO-CV and bootstrap optimism correction. Analyses incorporating NLR used the complete cohort (n = 51). Individual parameter independence and incremental clinical utility beyond NLR were not considered established by these models. No imputation was performed. Given the limited cohort size and the occurrence of only 13 deaths, the study was not powered to detect modest effects reliably, demonstrate biomarker independence from established COPD mortality determinants, or support the development of a multivariable prognostic model.

## 3. Results

Patient Characteristics: A total of 51 COPD patients were included in the study. The mean age was 65.76 ± 11.13 years (range 36–89 years); 23.5% (n = 12) were female and 76.5% (n = 39) were male. During the 24-month follow-up period, 13 patients died (25.5%) and 38 were alive (74.5%). Based on GOLD airflow limitation, 29 patients were GOLD 2, 19 were GOLD 3, and 3 were GOLD 4; no patient was GOLD 1. Based on GOLD A/B/E grouping, 2 patients were classified as group A, 30 as group B, and 19 as group E. The median partial BODE component score based on BMI, FEV1, and mMRC was 3 (IQR 2–4) among patients with available component data. Baseline demographic, clinical, functional, treatment, and outcome-related characteristics are summarized in Table 1.

Group Comparisons: In available-case analysis, arterial oxygen tension was lower among non-survivors than survivors (PaO2: 56.00 [IQR 49.90–58.70] vs. 59.20 [IQR 55.50–67.25] mmHg; *p* = 0.049). GOLD airflow limitation grade (*p* = 0.577), GOLD A/B/E group (*p* = 0.207), mMRC dyspnea score (*p* = 0.435), the partial BODE component score (*p* = 0.744), exacerbations in the previous year (*p* = 0.156), and hospitalizations in the previous year (*p* = 0.121) did not differ statistically between survival groups. Neutrophil percentages were numerically higher among non-survivors, whereas lymphocyte percentages were numerically lower, but these differences did not reach conventional statistical significance. NLR was higher in non-survivors but did not reach conventional statistical significance (median 4.19 [IQR 3.44–9.67] vs. 2.74 [IQR 1.93–5.60]; *p* = 0.056).

In unadjusted exploratory comparisons, CD45/SSC-defined lymphocyte-gate percentage and lymphocyte-gated CD138+ event percentage were nominally lower in patients who died. The median CD45/SSC-defined lymphocyte-gate percentage was 13.08% (IQR 10.13–14.11) in non-survivors versus 22.63% (IQR 14.70–27.80) in survivors (unadjusted *p* = 0.008). This variable represents the percentage of total acquired events falling within the lymphocyte region on the CD45/SSC dot plot and not CD45 expression intensity. Following source-report verification and correction of one transcription error, lymphocyte-gated CD138+ events were also nominally lower in non-survivors (median 0.07% [IQR 0.00–0.35] vs. 0.39% [IQR 0.16–1.46]; unadjusted *p* = 0.026). These CD138+ events were identified by single-parameter quadrant analysis within the lymphocyte gate and were not lineage-confirmed plasma cells. Within the hierarchical principal-variable family analysis, both findings retained significance (within-family q = 0.016 for CD45/SSC-defined lymphocyte-gate percentage and q = 0.026 for lymphocyte-gated CD138+ events); however, under a single pooled correction across all ten parameters, neither remained significant (Table 2).

Descriptive ROC Analysis of Key Lymphocyte-Gated Flow Cytometry Parameters: CD45/SSC-defined lymphocyte-gate percentage demonstrated an AUC of 0.749 (95% CI 0.585–0.913; unadjusted *p* = 0.003), and lymphocyte-gated CD138+ events demonstrated an AUC of 0.710 (95% CI 0.531–0.888; unadjusted *p* = 0.021). HLA-DR positivity showed weaker descriptive discrimination (AUC = 0.676, 95% CI approximately 0.490–0.862; *p* = 0.061) (Table 3). Post hoc internally derived thresholds, classification measures, and predictive values are provided only in Supplementary Table S1 and are not proposed as clinical or technical decision thresholds.

### Exploratory Signal-Aggregation Analysis and Post Hoc NLR Sensitivity Analyses

Because of the limited number of mortality events, the CD45/CD138 logistic analysis was undertaken only to assess the aggregated exploratory signal, not to develop a prediction model. In standard logistic regression using all 51 patients, the two-variable analysis yielded an apparent AUC of 0.858; LOO-CV AUC was 0.796 and bootstrap optimism-corrected AUC was 0.832 (Table 4). NLR was available for all 51 patients. NLR alone showed descriptive discrimination similar to the univariate NLR AUC (0.674). Adding CD45/SSC-defined lymphocyte-gate percentage and lymphocyte-gated CD138+ events to NLR improved model fit in a post hoc comparison (LR chi-square = 13.240, *p* = 0.001); however, this should not be interpreted as evidence of clinical incremental utility because the analysis was exploratory, event-limited, and not externally validated. When evaluated individually with NLR, CD45 did not retain nominal significance and CD138 was borderline. All effect estimates from the two-variable analysis should therefore be read as descriptive within-cohort estimates and not as evidence of independent prognostic effects.

Hierarchical multiplicity analysis: Multiplicity was assessed within two analytical families. Within the principal-variable family of the two biologically prioritized variables, both CD45/SSC-defined lymphocyte-gate percentage (unadjusted *p* = 0.008; within-family q = 0.016) and lymphocyte-gated CD138+ events (*p* = 0.026; within-family q = 0.026) retained statistical significance at q < 0.05. Within the secondary screening family of eight lymphocyte-gated parameters, no variable reached within-family significance, including HLA-DR positivity (*p* = 0.062; within-family q = 0.50) (Table 5). For transparency, when a single FDR adjustment was instead applied across all ten parameters jointly, no parameter retained significance (CD45 q = 0.081; CD138 q = 0.129); the principal-variable findings should therefore be interpreted in light of the limited event number and the absence of external validation. Thus, the statistical robustness of the two principal findings depended on the hierarchical family structure; the pooled analysis provides an important caution against interpreting the principal-variable family results as confirmatory evidence.

Multiplicity was assessed within two analytical families. Within the hierarchical principal-variable family analysis, CD45/SSC-defined lymphocyte-gate percentage and lymphocyte-gated CD138+ events retained significance (within-family q = 0.016 and 0.026); however, under a single pooled correction across all ten parameters, no parameter remained significant (CD45 q = 0.081; CD138 q = 0.129; HLA-DR q = 0.205). Consequently, the principal findings should be regarded as hypothesis-generating signals rather than multiplicity-robust associations.

## 4. Discussion

This single-center cohort evaluated associations between peripheral blood lymphocyte-gated flow cytometry-derived parameters and fixed 24-month all-cause mortality in stable outpatients with COPD. In unadjusted comparisons, non-survivors had lower CD45/SSC-defined lymphocyte-gate percentages, lower lymphocyte-gated CD138+ event percentages, and lower PaO2. Both principal variables retained significance within the principal-variable family, but neither remained significant after pooled FDR correction across all ten parameters. Accordingly, the findings identify candidates for prospective replication rather than validated prognostic biomarkers or a deployable prediction model.

COPD is a heterogeneous disease in which mortality risk is influenced not only by airflow limitation but also by symptom burden, exacerbation susceptibility, comorbidities, nutritional status, infection risk, and altered immune responses. In the present cohort, GOLD airflow limitation grade, GOLD A/B/E group, mMRC score, exacerbation history, hospitalization history, and the partial BODE component score did not differ statistically between survival groups, whereas PaO2 was lower among non-survivors. These clinical findings underscore that the flow cytometry-derived observations cannot be interpreted independently of established COPD prognostic determinants.

The most consistent exploratory observation in our cohort involved the CD45/SSC-defined lymphocyte-gate percentage. Importantly, this variable should not be interpreted as a measure of CD45 expression intensity. CD45 is a pan-leukocyte surface protein routinely used with side-scatter characteristics to distinguish major leukocyte populations during flow cytometric analysis [6]. In the present study, the recorded value represented the proportion of total acquired events located within the lymphocyte region on the CD45-versus-SSC plot. Thus, the lower values observed in non-survivors do not indicate reduced CD45 expression, CD45 downregulation, or a functional defect of CD45 signaling. Instead, they describe a smaller relative lymphocyte-gated compartment within the analyzed circulating leukocyte population.

Several biological interpretations may be considered for this observation. A reduced lymphocyte-gated percentage may reflect relative lymphopenia, neutrophil predominance, altered leukocyte distribution, chronic systemic inflammatory activity, or impaired adaptive immune reserve. Neutrophil percentages were numerically higher among non-survivors, although the between-group difference was not statistically significant. The inverse correlation with NLR and loss of nominal significance after NLR adjustment indicates overlap with systemic inflammatory imbalance rather than proven independent prognostic information. Larger studies incorporating absolute lymphocyte counts are needed to determine whether this gate-derived measure adds value beyond routine NLR [4,5].

Lower lymphocyte-gated CD138+ event percentages among non-survivors require cautious interpretation because CD138 positivity was determined by single-parameter quadrant analysis within the lymphocyte gate. CD138, or syndecan-1, is commonly associated with mature plasma cells and is used in plasma-cell immunophenotyping [7], but sequential lineage confirmation with CD19, CD38, and CD138 was not performed. The measured events were also low-frequency, and the association did not remain significant after pooled FDR correction. The finding therefore cannot be interpreted as depletion of circulating plasma cells or as an established mortality-associated biomarker.

One possible biological explanation is that reduced lymphocyte-gated CD138+ events reflect alterations in the circulating antibody-producing cell compartment or reduced humoral immune reserve. In COPD, low immunoglobulin concentrations and antibody deficiency have been associated with recurrent infections, frequent exacerbations, hospitalization, and unfavorable clinical courses [8,16]. A reduced humoral reserve could therefore increase susceptibility to respiratory infection and exacerbation-related systemic stress. However, because lineage-confirmed plasma-cell gating, serum immunoglobulin measurements, and longitudinal infection data were not available, this interpretation remains speculative and requires prospective testing.

HLA-DR positivity was evaluated as a secondary observation. Non-survivors had numerically higher lymphocyte-gated HLA-DR positivity than survivors, but neither the group comparison nor the descriptive ROC analysis met conventional statistical significance, and the signal did not persist after FDR correction. Because HLA-DR was measured within the overall lymphocyte gate rather than in lineage-defined subsets, no lineage-specific interpretation is appropriate. Prior COPD-related HLA-DR findings should therefore be considered biologically contextual rather than methodologically equivalent to the present total lymphocyte-gated HLA-DR measure [17].

The ROC analyses provide descriptive information about discrimination within this cohort but do not establish predictive utility. The reported thresholds were derived post hoc from the same dataset and are presented solely for descriptive transparency; they should not be interpreted as clinically applicable cutoffs without prospective external validation. This caution is especially important for the low-frequency CD138 threshold, which may be sensitive to analytical variability.

Multiplicity and confounding remain central to interpretation. Although both principal variables were significant within the principal-variable family, neither remained significant after pooled correction across all ten parameters. The post hoc comparison of NLR alone versus NLR plus both flow cytometry-derived variables suggested improved model fit, but the limited event count precluded robust multivariable adjustment and external validation. When evaluated individually with NLR, CD45 did not retain nominal significance and CD138 remained borderline. Thus, independence from established COPD prognostic factors and incremental prognostic value beyond routine inflammatory indices or established clinical predictors were not demonstrated.

Several conventional disease-related and lymphocyte-subset parameters, including FEV1, CRP, fibrinogen, CD3, CD4, CD5, CD8, CD19, CD26, and CD38, did not differ significantly between survivors and non-survivors. This does not imply that these variables are unimportant in COPD prognosis; the null findings may reflect limited statistical power, relatively homogeneous baseline characteristics, or the possibility that the observed mortality signal relates more closely to overall immune-cell distribution than to individual lymphocyte-subset percentages. The lack of discrimination by FEV1 also illustrates that spirometric impairment alone may not fully capture long-term risk in clinically heterogeneous COPD populations.

This study has several strengths. First, it evaluated a fixed and clinically relevant endpoint of 24-month all-cause mortality rather than a short-term surrogate outcome. Second, all participants had the same follow-up window, allowing clear assessment of survival status. Third, the analysis used transparent exploratory statistical procedures, including multiplicity adjustment and clearly labeled sensitivity analyses. Fourth, all flow cytometry-derived measurements were checked against archived original instrument reports before analysis, improving the reliability of variable classification and preventing inappropriate interpretation of CD45 as an MFI-based expression marker or CD138 as a confirmed plasma-cell measurement. Finally, the study explores routinely available laboratory data that may be feasible to incorporate into future prospective investigations without requiring highly complex sampling procedures.

Several limitations remain. This was a single-center study of 51 patients with 13 deaths, which limited statistical precision and precluded robust simultaneous adjustment for established COPD prognostic factors. The full BODE index could not be calculated because the 6-min walk distance was unavailable, and the reported partial BODE component score should not be interpreted as the validated BODE index. The flow cytometry protocol was not lineage-confirmed: CD45 was recorded as a gate-derived lymphocyte percentage, CD138 positivity was not confirmed using sequential plasma-cell gating, and HLA-DR was not examined within predefined immune-cell subsets. In addition, the principal findings were not retained after pooled correction across all ten parameters. These limitations preclude interpretation of the observed associations as validated biomarkers, independent prognostic effects, or clinically applicable cutoffs.

Future studies should evaluate these observations in larger prospective multicenter cohorts with adequate adjustment for established COPD prognostic factors. Standardized protocols should incorporate absolute leukocyte and lymphocyte counts, lineage-confirmed plasma-cell identification, and HLA-DR analysis within predefined T-cell, B-cell, and monocyte subsets. Parallel assessment of serum immunoglobulins, respiratory infections, exacerbations, hospitalizations, and longitudinal immune changes could clarify the biological relevance of the CD138-related signal. Clinical application would additionally require analytical reproducibility, standardized pre-analytical handling, independent external validation of discrimination and calibration, and demonstration of meaningful incremental value beyond established COPD risk measures.

## 5. Conclusions

In this small exploratory COPD cohort with fixed 24-month follow-up, lower CD45/SSC-defined lymphocyte-gate percentage and lower lymphocyte-gated CD138+ event percentage were observed among non-survivors, while PaO2 was also lower. These within-cohort associations should be regarded solely as hypothesis-generating signals. Although both principal variables retained significance within the hierarchical principal-variable family analysis, neither finding survived pooled correction across all ten flow cytometry-derived parameters. Robust independence from established COPD prognostic factors and incremental prognostic value beyond routine inflammatory indices or established clinical predictors were not demonstrated. The findings do not support clinical decision thresholds or biomarker use and require prospective replication, adequate multivariable adjustment, standardized lineage-specific flow cytometry, and independent external validation.

## Figures and Tables

**Table 1:** Demographic, clinical, functional, treatment, and outcome-related baseline characteristics according to 24-month survival status.

Parameter	Survivors	Non-Survivors	*p* Value
Male sex, n/N (%)	29/38 (76.3)	10/13 (76.9)	1.000
Age, years	66.00 (59.00–73.00)	69.00 (59.00–76.00)	0.314
BMI, kg/m^2^	25.46 (24.31–30.39) [n = 33]	25.71 (24.78–27.06) [n = 13]	0.971
Smoking status, n/N (%)	Current: 15/38 (39.5) Former: 15/38 (39.5) Never: 8/38 (21.1)	Current: 5/13 (38.5) Former: 6/13 (46.2) Never: 2/13 (15.4)	1.000
Smoking history, pack-years	25.00 (20.00–30.00) [n = 30]	25.00 (20.00–30.00) [n = 11]	0.892
FEV1, % predicted	52.00 (42.25–60.00)	50.00 (42.00–58.00)	0.829
GOLD airflow limitation grade, n/N (%)	GOLD 2: 21/38 (55.3) GOLD 3: 14/38 (36.8) GOLD 4: 3/38 (7.9)	GOLD 2: 8/13 (61.5) GOLD 3: 5/13 (38.5) GOLD 4: 0/13 (0.0)	0.577
PaO2, mmHg	59.20 (55.50–67.25) [n = 36]	56.00 (49.90–58.70) [n = 13]	0.049
mMRC dyspnea score	3.00 (2.00–3.00)	3.00 (2.00–4.00)	0.435
GOLD A/B/E group, n/N (%)	A: 1/38 (2.6) B: 25/38 (65.8) E: 12/38 (31.6)	A: 1/13 (7.7) B: 5/13 (38.5) E: 7/13 (53.8)	0.207
Partial BODE component score (BMI + FEV1 + mMRC; 0–7)	3.00 (2.00–4.00) [n = 33]	3.00 (2.00–5.00) [n = 13]	0.744
Inhaled corticosteroid-containing therapy, n/N (%)	35/38 (92.1)	12/13 (92.3)	1.000
LABA/LAMA-containing maintenance therapy, n/N (%)	38/38 (100.0)	13/13 (100.0)	1.000
Long-term oxygen therapy, n/N (%)	9/38 (23.7)	2/13 (15.4)	0.706
Exacerbations in previous year	0.00 (0.00–1.00)	1.00 (0.00–2.00)	0.156
Hospitalizations in previous year	0.00 (0.00–0.75)	0.00 (0.00–1.00)	0.121
Diabetes mellitus, n/N (%)	3/38 (7.9)	3/13 (23.1)	0.154
Hypertension, n/N (%)	4/38 (10.5)	3/13 (23.1)	0.356
Coronary artery disease, n/N (%)	4/38 (10.5)	1/13 (7.7)	1.000

Data are presented as n/N (%) or median (IQR). BMI: body mass index; FEV1: forced expiratory volume in 1 s; LABA/LAMA: long-acting beta-2 agonist/long-acting muscarinic antagonist; mMRC: modified Medical Research Council; PaO2: arterial oxygen tension. Analyses used available data; missing denominators are shown where applicable. Smoking-status categories were mutually exclusive (current, former, or never smoker). Former smokers were participants who were not current smokers but had a previous smoking history. The smoking-status *p* value was calculated using the Fisher–Freeman–Halton exact test. Pack-year data were available for ever-smokers (30 survivors and 11 non-survivors).

**Table 2:** Routine inflammatory indices and lymphocyte-gated flow cytometry parameters according to 24-month survival status.

Variable	Presentation	Non-Survivors	Survivors	*p* Value
Neutrophils (%)	Median (IQR)	72.30 (68.20–79.50)	65.45 (55.88–78.28)	0.117
Lymphocytes (%)	Median (IQR)	18.00 (8.00–19.80)	23.40 (14.03–30.40)	0.053
NLR	Median (IQR)	4.19 (3.44–9.67)	2.74 (1.93–5.60)	0.056
CRP (mg/L)	Median (IQR)	5.00 (0.80–26.01)	5.35 (2.67–23.18) [n = 32]	0.950
Fibrinogen (mg/dL)	Median (IQR)	450.00 (412.00–513.00)	441.50 (378.50–541.50)	0.991
CD3 (%)	Median (IQR)	69.83 (65.27–76.90)	68.98 (66.27–74.06)	0.770
CD4 (%)	Median (IQR)	44.50 (40.59–52.16)	46.55 (39.22–51.85)	0.888
CD5 (%)	Median (IQR)	69.96 (64.29–77.20)	69.41 (66.33–72.94)	0.905
CD8 (%)	Median (IQR)	35.44 (30.69–38.31)	33.38 (28.86–42.99)	0.770
CD19 (%)	Median (IQR)	7.90 (6.39–9.94)	9.48 (6.66–12.74)	0.589
CD26 (%)	Median (IQR)	34.48 (27.57–36.98)	35.63 (31.30–42.01)	0.222
CD38 (%)	Median (IQR)	23.35 (19.00–27.93)	22.90 (18.52–29.07)	0.754
CD45/SSC-defined lymphocyte-gate percentage (%)	Median (IQR)	13.08 (10.13–14.11)	22.63 (14.70–27.80)	0.008
Lymphocyte-gated CD138+ events (%)	Median (IQR)	0.07 (0.00–0.35)	0.39 (0.16–1.46)	0.026
HLA-DR positivity within lymphocyte gate (%)	Median (IQR)	25.31 (19.45–29.11)	20.34 (16.46–25.31)	0.062

*Data are presented as median (IQR).* After source verification, NLR was available for all 51 patients (13 non-survivors and 38 survivors). One CD138 value in the source dataset was corrected against the original instrument report before analysis. Unadjusted *p* values are shown; multiplicity-adjusted significance was assessed within two analytical families and is reported in the hierarchical multiplicity analysis below.

**Table 3:** Descriptive ROC AUC analysis of selected flow cytometry-derived parameters for fixed 24-month mortality.

Variable	AUC	95% CI	Unadjusted *p* Value
CD45/SSC-defined lymphocyte-gate percentage (%)	0.749	0.585–0.913	0.003
Lymphocyte-gated CD138+ events (%)	0.710	0.531–0.888	0.021
HLA-DR positivity within lymphocyte gate (%)	0.676	0.490–0.862	0.061

AUC: area under the receiver operating characteristic curve; CI: confidence interval. AUC analyses are descriptive and use unadjusted *p* values. Multiplicity-adjusted group-comparison results are reported in the hierarchical multiplicity analysis below. Full post hoc threshold and classification estimates are presented in Supplementary Table S1 only.

**Table 4:** Exploratory signal-aggregation analysis and descriptive sensitivity analyses.

Analysis/Model	Available n/Deaths	Effect Estimate	*p* Value/Performance
Exploratory two-variable logistic analysis: CD45/SSC lymphocyte-gate %	51/13	OR 0.869 (95% CI 0.783–0.964)	*p* = 0.008; apparent AUC 0.858
Exploratory two-variable logistic analysis: lymphocyte-gated CD138+ events	51/13	OR 0.227 (95% CI 0.054–0.946)	*p* = 0.042
LOO-CV descriptive assessment of two-variable analysis	51/13	LOO-CV AUC 0.796	Internal discrimination assessment
Bootstrap descriptive assessment of two-variable analysis	51/13	Optimism-corrected AUC 0.832	1000 stratified resamples; 999 successful fits
NLR discrimination for mortality	51/13	AUC 0.674	Full-cohort descriptive analysis
CD45/SSC lymphocyte-gate % versus NLR	51/13	Spearman rho = −0.533	*p* < 0.001
NLR + CD45/SSC lymphocyte-gate %	51/13	CD45 OR 0.939 (95% CI 0.858–1.028)	*p* = 0.172
NLR + lymphocyte-gated CD138+ events	51/13	CD138 OR 0.157 (95% CI 0.023–1.064)	*p* = 0.058
NLR + CD45 + CD138 versus NLR alone (full-cohort NLR model)	51/13	LR chi-square = 13.240, df = 2	*p* = 0.001; full-cohort model AUC 0.863; not evidence of clinical incremental utility

AUC: area under the receiver operating characteristic curve; CI: confidence interval; LOO-CV: leave-one-out cross-validation; LR: likelihood-ratio test; NLR: neutrophil-to-lymphocyte ratio; OR: odds ratio. The two-variable analysis was not intended for clinical prediction model development or validation. Cross-validation, bootstrap optimism correction, and NLR-adjusted analyses were descriptive sensitivity analyses. The global NLR model comparison is not evidence of clinical incremental utility. The reported odds ratios are descriptive within-cohort estimates and are not evidence of independent prognostic effects. The apparent AUC of 0.858 and the optimism-corrected AUC of 0.832 refer to the two-variable CD45/CD138 analysis in the full cohort. The AUC of 0.863 refers to the NLR + CD45 + CD138 sensitivity model in the full cohort and should not be compared as an externally validated performance estimate.

**Table 5:** Hierarchical multiplicity adjustment of flow cytometry-derived parameters. q values were computed by Benjamini–Hochberg adjustment separately within two analytical families: a principal-variable family including CD45/SSC-defined lymphocyte-gate percentage and lymphocyte-gated CD138+ events, and a secondary screening family including the remaining eight parameters. Both principal variables retained significance within the principal-variable family (within-family q = 0.016 and 0.026); no secondary-family parameter reached within-family significance. For reference, under a single pooled adjustment across all ten parameters, no parameter retained significance (CD45 q = 0.081; CD138 q = 0.129; HLA-DR q = 0.205).

Flow Cytometry-Derived Parameter	Unadjusted *p* Value	Within-Family FDR-Adjusted q Value
CD45/SSC-defined lymphocyte-gate percentage	0.008	0.016
Lymphocyte-gated CD138+ events	0.026	0.026
HLA-DR positivity within lymphocyte gate	0.062	0.496
CD26	0.222	0.888
CD19	0.589	0.905
CD38	0.754	0.905
CD3	0.770	0.905
CD8	0.770	0.905
CD4	0.888	0.905
CD5	0.905	0.905

## Data Availability

The data presented in this study are available from the corresponding author upon reasonable request.

## Supplementary Materials

The following supporting information can be downloaded at: https://www.mdpi.com/article/10.3390/jcm15145333/s1, Table S1: Post Hoc Internally Derived ROC Threshold and Classification Estimates.
