# Supplementary material for: Peripheral Blood Lymphocyte-Gated Flow Cytometry Parameters and 24-Month Mortality in COPD: An Exploratory Cohort Study
Source: J Clin Med. 2026 Jul 8;15(14):5333. doi: 10.3390/jcm15145333 (PMC13412794; doi:10.3390/jcm15145333)
Supplement: Supplementary file 1 [file jcm-15-05333-s001.zip › jcm-4403167-supplementary.pdf]

Supplementary Table S1. Post Hoc Internally Derived ROC Threshold and Classification Estimates

Associated manuscript: Peripheral Blood Lymphocyte-Gated Flow Cytometry Parameters and 24-Month Mortality in COPD: An Exploratory Cohort Study

The estimates below were derived post hoc from the same small exploratory cohort used for analysis. They are reported for descriptive transparency only and are not proposed for clinical, prognostic, diagnostic, or technical classification. These estimates should not be interpreted as clinically applicable cutoffs without prospective external validation.

Variable	Post hoc cut-off	Sensitivity (%)	Specificity (%)	PPV (%)	NPV (%)	AUC (95% CI)	Unadjusted p
CD45/SSC-defined lymphocyte-gate percentage (%)	≤14.11	76.9	76.3	52.6	90.6	0.749 (0.585-0.913)	0.003
Lymphocyte-gated CD138+ events (%)	≤0.05	46.2	92.1	66.7	83.3	0.710 (0.531-0.888)	0.021
HLA-DR positivity within lymphocyte gate (%)	≥26.29	46.2	86.8	54.5	82.5	0.676 (0.490-0.862)	0.061

Abbreviations: AUC, area under the receiver operating characteristic curve; CI, confidence interval; NPV, negative predictive value; PPV, positive predictive value. All ROC cut-offs were internally derived post hoc descriptive values. For lymphocyte-gated CD138+ events, the ≤0.05% threshold corresponds to approximately 10 positive events in the sample with the lowest observed lymphocyte-gate event count and should not be interpreted as technically validated or clinically applicable without prospective external validation.
